# Immunomodulatory piezoelectric master electrospun membranes for pelvic floor repair

**DOI:** 10.1186/s12951-026-04582-0

**Published:** 2026-05-22

**Authors:** Xiaohan Fu, Haibo Chen, Wei Geng, Xiao Hou, Wenlan Xing, Min Cui, Xiumei Mo, Xiuyun Li

**Affiliations:** 1 Infection and Microbiology Research Laboratory for Women and Children, Shandong Provincial Maternal and Child Health Care Hospital Affiliated to Qingdao University, Jinan, 250014 P. R. China; 2 Department of Plastic Surgery, Shandong Provincial Maternal and Child Health Care Hospital Affiliated to Qingdao University, Jinan, 250014 P. R. China; 3 Department of General Surgery, Shandong Provincial Maternal and Child Health Care Hospital Affiliated to Qingdao University, Jinan, 250014 P. R. China; 4 Department of Traditional Chinese Medicine, Shandong Provincial Maternal and Child Health Care Hospital Affiliated to Qingdao University, Jinan, 250014 P. R. China; 5 Diagnosis and Treatment Center for Pelvic Floor Disease, Shandong Provincial Maternal and Child Health Care Hospital Affiliated to Qingdao University, Jinan, 250014 P. R. China; 6 State Key Laboratory for Modification of Chemical Fibers and Polymer Materials, Shanghai Engineering Research Center of Nano-Biomaterials and Regenerative Medicine, College of Biological Science and Medical Engineering, Donghua University, Shanghai, 201620 P. R. China

**Keywords:** Pelvic organ prolapse, Electrospun, Piezoelectric, Immunomodulatory

## Abstract

Pelvic floor dysfunction (PFD) in women is characterized primarily by the functional degeneration of pelvic support structures, with pelvic organ prolapse (POP) being a common clinical manifestation. Current materials for repairing POP are limited by suboptimal mechanical performance and inadequate anti-inflammatory and antibacterial capabilities. Herein, a immunomodulatory piezoelectric master electrospun membrane is innovatively designed to provide integrated mechanical support, piezoelectric responsiveness, and immune microenvironment regulation. Electrospun membranes with uniformly loaded zinc oxide (ZnO) nanoparticles enabled the sustained release of Zn²⁺ for more than 20 days and achieved a piezoelectric coefficient (*d*_*33*_) of 5.34 pm/V. The voltage output remained stable under mechanical stimulation cycles, and the ultimate tensile strength could reach 12.11 MPa while maintaining sufficient ductility. In vitro, the immunomodulatory piezoelectric master electrospun membrane exhibited excellent biocompatibility, significantly promoted tube formation by human umbilical vein endothelial cells (HUVECs), and upregulating the expression of IL-4 and IL-10. Moreover, it effectively inhibited inflammatory cell infiltration, promoted orderly collagen deposition, stimulated angiogenesis and accelerated tissue repair when used in vivo to treat abdominal wall muscle defects in rats. Therefore, immunomodulatory piezoelectric master electrospun membrane provides a promising approach for advanced soft tissue regeneration with broad clinical significance.

**Graphical Abstract:**

**Figure:**
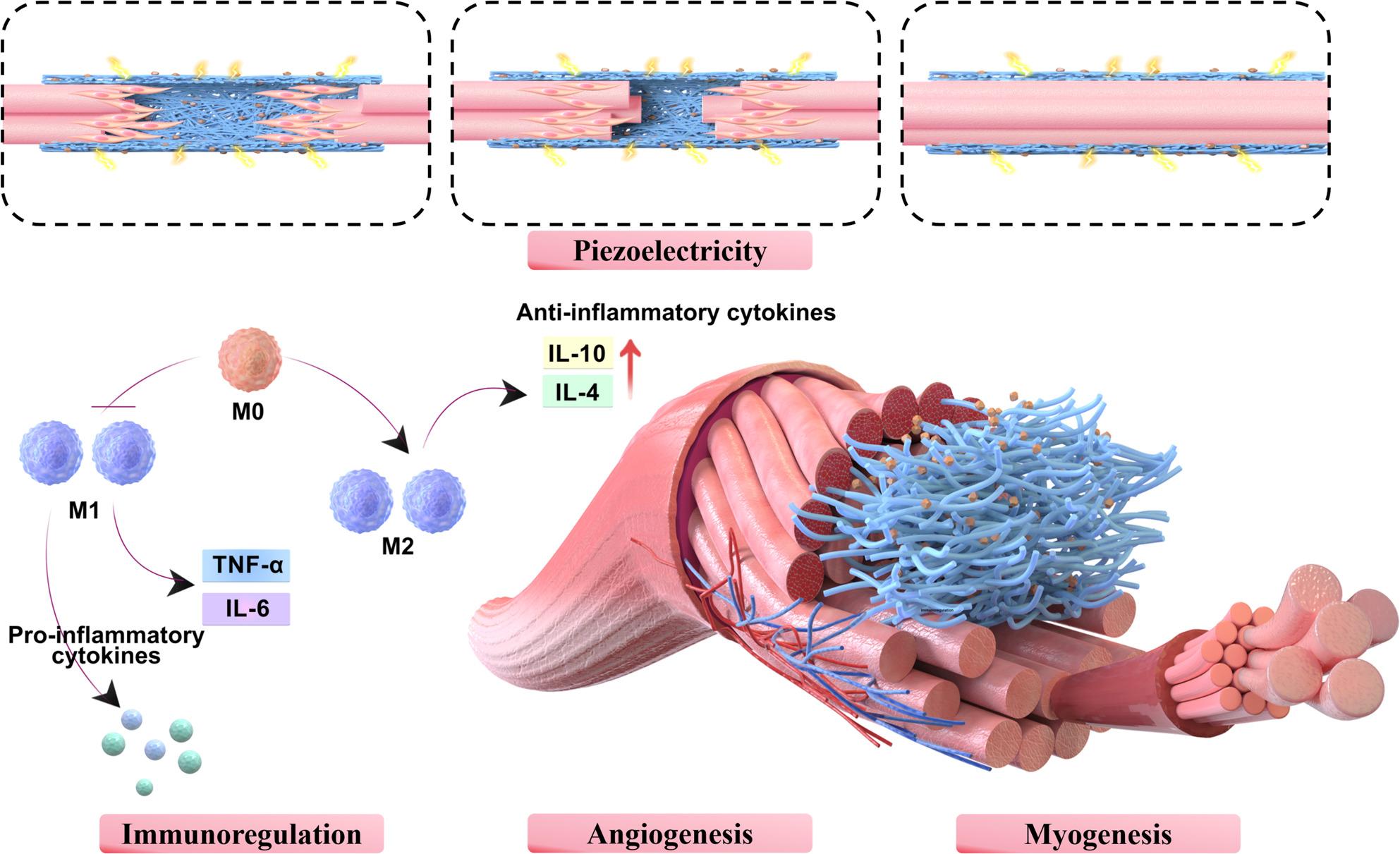
The immunomodulatory piezoelectric master electrospun membrane possesses excellent piezoelectric, anti-inflammatory and antibacterial properties. They have achieved the transformation from passive support to active response, and from single function to multiple synergistic effects, thereby enabling the repair of soft tissues

**Supplementary Information:**

The online version contains supplementary material available at 10.1186/s12951-026-04582-0.

## Introduction

Female pelvic floor dysfunction (PFD) is a significant public health concern that profoundly affects women’s quality of life. Its core pathological feature involves functional deterioration of pelvic floor support structures, such as muscles, fascia, and ligaments, due to pregnancy-related injury, ageing, or abnormal mechanical loading [1]. Clinically, PFD primarily manifests as pelvic organ prolapse (POP) and stress urinary incontinence [2, 3]. Particularly in POP, weakened pelvic floor support tissues lead to organ descent and displacement, resulting in functional abnormalities that severely impact patients’ quality of life [4]. Current clinical treatment strategies for POP are primarily categorized into nonsurgical and surgical approaches. Nonsurgical therapies, such as electrical stimulation therapy, utilize exogenous electrical pulses to stimulate pelvic floor nerves, inducing passive muscle contractions [5, 6]. While these therapies can temporarily improve muscle tone and pelvic function, they cannot repair ruptured damaged connective tissue and often require long-term adherence [7]. In contrast, surgical intervention remains the primary treatment for moderate to severe POP and involves the implantation of synthetic mesh or biological grafts to reinforce weakened areas of the pelvic floor [8–10]. However, the currently reported materials used for repairing POP exhibit certain limitations to varying degrees, and an ideal implantable material has yet to be identified in clinical settings. On the one hand, pelvic floor tissues endure periodic mechanical stress during daily activities such as urination and defecation, necessitating implantable materials that possess both high tensile strength to withstand intra-abdominal pressure and sufficient elasticity to accommodate dynamic tissue movement [11]. On the other hand, the implantation of materials via vaginal or abdominal approaches can easily cause chronic inflammation and infections after surgery [12]. Inflammation may lead to fibrosis and mesh erosion, whereas infections can trigger acute pelvic sepsis, often necessitating surgical removal of the implantable materials [13]. Thus, these unmet clinical needs have catalysed a paradigm shift in the design of POP repair materials, from passive structural scaffolds to functionally integrated responsive systems.

In recent years, the “active responsiveness” of biomaterials has emerged as an innovative strategy to overcome the limitations of traditional passive implants. Among these materials, piezoelectric materials stand out because of their unique advantages. They can convert physiological mechanical stimuli, such as muscle contractions and joint movements, into endogenous electrical signals, making them an ideal platform for activating tissue regeneration pathways [14]. Endogenous bioelectric signals play critical roles in regulating cell proliferation, differentiation, and extracellular matrix synthesis. For instance, in wound healing applications, piezoelectric materials generate localized electric fields that enhance fibroblast migration, promote endothelial tubulogenesis, and upregulate collagen-related gene expression [15]. Currently, piezoelectric materials have been applied in various fields such as bone tissue engineering (for promoting bone cell mineralization) and nerve repair (for guiding the growth of nerve axons) [16, 17]. In recent years, biodegradable piezoelectric materials (such as poly-L-lactic acid, polyvinylidene fluoride, etc.) have made significant progress in the field of tissue regeneration. Researchers have successfully applied them to bone defect repair, tendon healing, and cardiac tissue engineering, effectively promoting cell directed differentiation and functional tissue regeneration through endogenous electrical signals [18–21]. However, in the field of POP repair, the application of piezoelectric performance materials is still lacking, and the existing piezoelectric materials, although capable of achieving mechanical-electrical signal conversion, have obvious limitations and are difficult to be directly applied to POP repair: (1) their mechanical properties are significantly limited because balancing their tensile strength and ductility is difficult, which leads to fatigue failure under repeated pelvic loading; (2) they lack intrinsic biological regulation capabilities, which limits their ability to modulate postoperative inflammation and prevent infections. Current surgical approaches for POP repair commonly involve transvaginal or transabdominal mesh implantation, both of which carry risks of postoperative inflammation and infection; (3) finally, their biocompatibility is poor and they trigger local immune responses, which compromises their long-term safety [22]. Consequently, the development of multifunctional repair materials that integrate piezoelectric activity, mechanical adaptability, and anti-inflammatory/antibacterial properties represents a pivotal frontier in overcoming current challenges in POP treatment.

Common biomaterials used for soft tissue repair include hydrogels, microspheres, and fibrous membranes. Among these, fibrous membranes prepared by electrospinning have garnered significant attention in tissue engineering because of their ability to precisely mimic the structural characteristics and surface morphology of the natural extracellular matrix (ECM) [23, 24]. Their ability to simulate the microenvironment creates favourable conditions for cell adhesion, proliferation, and differentiation [25]. For instance, the polycaprolactone (PCL)/gelatine (Gel) composite nanofibres developed by Xiang et al. exhibit a porosity exceeding 85%, significantly enhancing fibroblast infiltration and growth capacity [26]. Niu et al. incorporated hydroxyapatite nanoparticles to improve mechanical robustness, thereby increasing the durability of the scaffold under load-bearing conditions [27]. Nevertheless, traditional electrospun membranes have functional limitations: first, they primarily serve as passive scaffolds that cannot respond to physiological mechanical signals and are thus unable to utilize endogenous bioelectrical signals for regenerative stimulation; second, they exhibit minimal intrinsic anti-inflammatory activity; instead, they rely on passive biocompatibility to mitigate foreign body reactions, which is insufficient for controlling postoperative inflammatory cascades; and third, their antibacterial function is typically achieved through antibiotic incorporation, which may induce antibiotic resistance and diminish efficacy as antibiotic concentrations decrease over time. These shortcomings hinder the clinical translation of electrospun membranes in POP repair, underscoring the urgent need for innovative, multifunctional designs that transcend the boundaries of passive structural support.

**Scheme 1:**
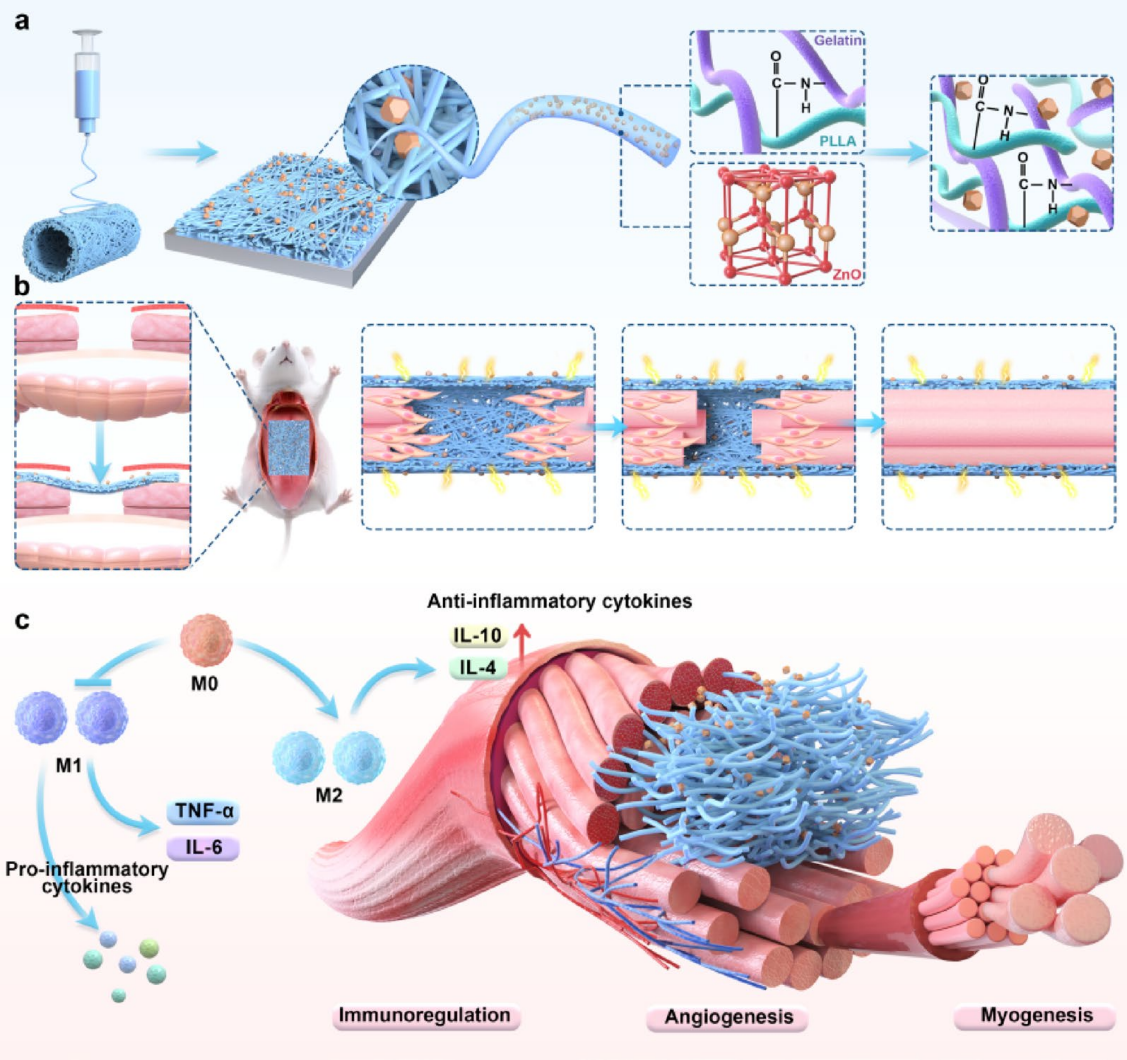
Schematic illustration. **a**) Preparation of a immunomodulatory piezoelectric master electrospun membrane. **b**) Piezoelectric master electrospun membranes generate piezoelectric signals that stimulate myogenic cell proliferation, thereby promoting abdominal wall muscle regeneration. **c**) Piezoelectric master electrospun membrane for pelvic floor repair

Poly-L-lactic acid (PLLA) is a biocompatible and biodegradable polymer that has garnered significant attention in the field of piezoelectric materials in recent years because of its excellent physicochemical properties and ability to generate endogenous electrical signals in response to mechanical stimulation [28]. Moreover, zinc oxide (ZnO) nanoparticles, which are potent agents with inherent anti-inflammatory and antibacterial activities, can modulate immune responses and inhibit microbial growth in the absence of traditional antibiotics [29]. Unlike the current research on piezoelectric materials, the core of this study lies in the first time that the “piezoelectric - anti-inflammatory - antibacterial” triple functions have been synergistically integrated. In this study, an immunomodulatory piezoelectric master electrospun membrane was innovatively constructed by cospinning PLLA, Gel, and ZnO (Scheme 1). First, the piezoelectric performance, mechanical properties, and Zn^2+^ release profiles of the electrospun membranes were characterized. Their biocompatibility and in vitro anti-inflammatory and antibacterial efficacy were subsequently validated. Finally, the in vivo regenerative capacity of the membranes was tested in a rat model of an abdominal wall muscle defect. This study aims to provide a new type of “functionally coordinated and physiologically compatible” tissue engineering material for the treatment of POP. The conceptual innovation lies in the transformation from passive support to active response, and from a single function to multiple synergies. At the same time, it provides new ideas for the multifunctional design of soft tissue repair materials.

## Experimental section

### Materials

Gel, polylactic acid (PLA), and poly-L-lactic acid (PLLA) were obtained from Jinan Daigang Bioengineering Co., Ltd. ZnO was supplied by Shanghai Titan Technology Co., Ltd. Hexafluoroisopropanol (HFIP) was purchased from Shanghai Maclin Co., Ltd. Cell culture reagents were sourced from Gibco. Additional specialized reagents used in the experiments are described separately.

### Preparation of the PG-ZnO membrane

PLA (1.3 g) and PLLA (1.0 g) were weighed separately. Each polymer was mixed with 0.3 g of Gel, dissolved in 10 mL of HFIP, and magnetically stirred until fully dissolved. Two electrospinning solutions were prepared: the NPG electrospinning solution (PLA/Gel system) and the PG electrospinning solution (PLLA/Gel system). To prepare the PG-ZnO composite electrospinning solution, 3 mg、5 mg and 7 mg of ZnO powder was added to 10 mL of PG solution respectively and magnetically stirred until the ZnO particles were uniformly dispersed. Electrospun NPG, PG, and PG-ZnO membranes were prepared using an electrospinning device (at a voltage of 17 kV and a feed rate of 0.25 mm/min).

### Characterization of membranes

The internal morphologies of the three groups of membranes were examined with scanning electron microscopy (SEM; ZEISS Sigma 300, Germany). Elemental composition was analysed with an integrated energy dispersive spectrometer (EDS mapping) of the SEM. The average diameter and porosity of the fibres were quantified using ImageJ software. The internal microstructures of the fibres were further observed with a transmission electron microscope (TEM, JEOL JEM-F200, Japan) after they were collected on carbon-coated copper grids. The contact angles were measured with a contact angle analyser (Alpha-S, China). The chemical structures of the membranes were analysed with Fourier transform infrared (FTIR) spectroscopy (Thermo Fisher Scientific Nicolet iS20, USA) over a spectral range of 4000–800 cm⁻¹. Mechanical properties were assessed by cutting the membranes into 15 × 10 mm rectangular samples and performing tensile tests at a crosshead speed of 20 mm/min and repeated 5 times with an electronic universal testing machine (INSTRON 5982, USA).

Cut the 5 mg sample into small pieces and weigh them (W_0_). Then immerse them in 2 mL of PBS. Take the samples out at 0.5 h, 1 h, 2 h, 4 h, 24 h and 72 h, remove the excess water and weigh the samples again (W_1_) [30]. The swelling percentage of the membrane was calculated using the following formula:

1 $$\rm Swelling rate (\%)= (W_{1} - W_{0}) / M_{0}\times100\% $$

The degradation experiment of the membranes were conducted in vitro under simulated body fluid environment. The membranes were immersed in PBS at 37 °C for 7, 14 and 21 days. The initial weight (W_0_) of the sample before immersion and the weight (W_1_) after drying treatment after immersion were recorded [31]. Formula is as follows:

2 $$\rm Degradation rate (\%) = (W_{0} - W_{1}) / W_{0}\times100\% $$

Three groups of membranes (2 × 2 cm²) were sterilized and then immersed in centrifuge tubes containing 5 mL of phosphate-buffered saline (PBS). The tubes were then sealed and incubated in a 37 °C water bath. At predetermined time points (1 h, 8 h, 1 d, 4 d, 7 d, 14 d, and 21 d), the supernatant was collected and replaced with fresh PBS. The concentrations of released Zn²⁺ were quantified using inductively coupled plasma‒mass spectrometry (ICP‒MS, USA).

### Piezoelectric properties of membranes

The surface microstructures and piezoelectric responses of the membranes were evaluated using an atomic force microscope (AFM, Bruker Dimension Icon, Germany) equipped with a piezoelectric force microscopy (PFM) module. The static piezoelectric coefficient *d₃₃* was measured using an oil-bath corona composite piezoelectric polarization device (FJH-3). A 2 mm×2 mm gold electrode was deposited on the sample surface, matching the probe size. Measurements were conducted in a top-to-bottom configuration. A 10 cm×10 cm membrane was fixed on the pressure sensor, and different pressures were applied to the membrane. The output voltage was then reflected and recorded in real time by the oscilloscope (Keysight DSOX3024T).

### In vitro experiments

In vitro cell experiments were performed using human umbilical vein endothelial cells (HUVECs) and RAW 264.7 cells cultured in high-glucose DMEM.

#### Preparation of extract liquids

In accordance with international ISO standards, the sterilized membranes were immersed in basal medium for 3 days (at a concentration of 0.1 g/mL). Foetal bovine serum (FBS; Gibco) was then added to the solution, and finally, the solution was filtered to obtain the extract liquids of the membranes.

#### Cell adhesion

The three groups of membranes were cut into appropriate sizes, sterilized, and placed in 48-well plates. HUVECs were seeded onto sterilized membranes at a density of 3.0 × 10⁴ cells/membrane. After 7 days of culture, cell adhesion and infiltration were evaluated using SEM, haematoxylin and eosin (H&E) staining, and DAPI/phalloidin immunofluorescence staining.

#### Assessment of tube formation

HUVECs were seeded at a density of 5.0 × 10⁴ cells/well in a confocal plate. After cell attachment, the culture medium was replaced with the extract liquids of the membranes. Following 4 h of incubation, tube formation was assessed using a Live/Dead Staining Kit (Beyotime, China), and images were captured under a fluorescence microscope (Olympus, Tokyo, Japan). Tube formation was quantified using ImageJ software.

#### Biocompatibility

HUVECs and RAW 264.7 cells were seeded at a density of 3.0 × 10³ cells/well in a 96-well plate. After cell attachment, the culture medium was replaced with the material extract liquids. On Days 1, 3, and 5, CCK-8 working solution (Beyotime, China) was added to assess cell viability by measuring the optical density at 450 nm using a microplate reader.

#### Anti-inflammatory activity

RAW 264.7 cells were seeded at a density of 1 × 10⁵ cells/well in a 24-well plate. After cell attachment, the culture medium was replaced with the extract liquids, and the cells were incubated for 24 h. The medium was subsequently replaced with extract liquids containing 100 ng/mL lipopolysaccharide (LPS) for another 24 h of incubation. Upon completion of the incubation, the supernatant was collected for further analysis.

#### Antibacterial test

*Escherichia coli* and *Staphylococcus aureus* were cultured overnight on Mueller‒Hinton agar plates in a shaking incubator at 35 °C. The bacterial inoculum was adjusted to a concentration of 10⁵ CFU/mL, and 100 µL of the suspension was spread onto the surface of sterile membranes, which were then incubated for 24 h. Following incubation, the bacterial-coated membranes were transferred into 2 mL of sterile PBS and vortexed to dislodge the bacteria. The PBS suspensions were centrifuged at 800 rpm for 15 min. The pellets were resuspended in sterile PBS after centrifugation and subjected to 10-fold and 100-fold serial dilutions. Five microlitres of the ustock solution, 10-fold diluted solution, and 100-fold diluted solution were spotted onto Mueller–Hinton agar plates. After 24 h of incubation, the bacterial colonies were photographed and quantitative analysis was performed on the colonies grown in the ustock solution.

### In vivo repair of abdominal wall muscle defects

The in vivo experimental protocol was approved by the Shandong Maternal and Child Health Hospital Laboratory Animal Ethics Committee (No. 2024-088) and was conducted in compliance with institutional guidelines for animal care and use. Eight-week-old female Sprague‒Dawley (SD) rats were acclimatized for one week with unrestricted access to food and water. The rats were then randomly divided into four groups: the control group, the NPG group, the PG group, and the PG-ZnO group. Under anaesthesia and aseptic conditions, the abdominal area was disinfected [32]. A full-thickness abdominal wall defect (1 cm in diameter) was created by excising muscle tissue while preserving the peritoneum. The electrospun membranes were implanted into the defect sites, after which the abdominal skin was sutured. To evaluate the inflammatory response, collagen deposition, and angiogenesis in the injury area, histological analysis was performed using H&E staining, Masson’s trichrome staining, and immunofluorescence staining (type I and III collagen, α-SMA, CD 68, iNOS, CD 206, and CD31). Tissue samples were collected on Days 14 and 28.

### Statistical analysis

All the experimental data were analysed using GraphPad Prism 10. Statistical differences among multiple groups were evaluated by one-way ANOVA. The data are expressed as the mean ± standard deviation. Differences were considered statistically significant at *p* < 0.05 and *p* < 0.01, denoted as *p < 0.05 and **p < 0.01, respectively.

## Results and discussion

### Preparation and characterization of membranes

**Fig. 1:**
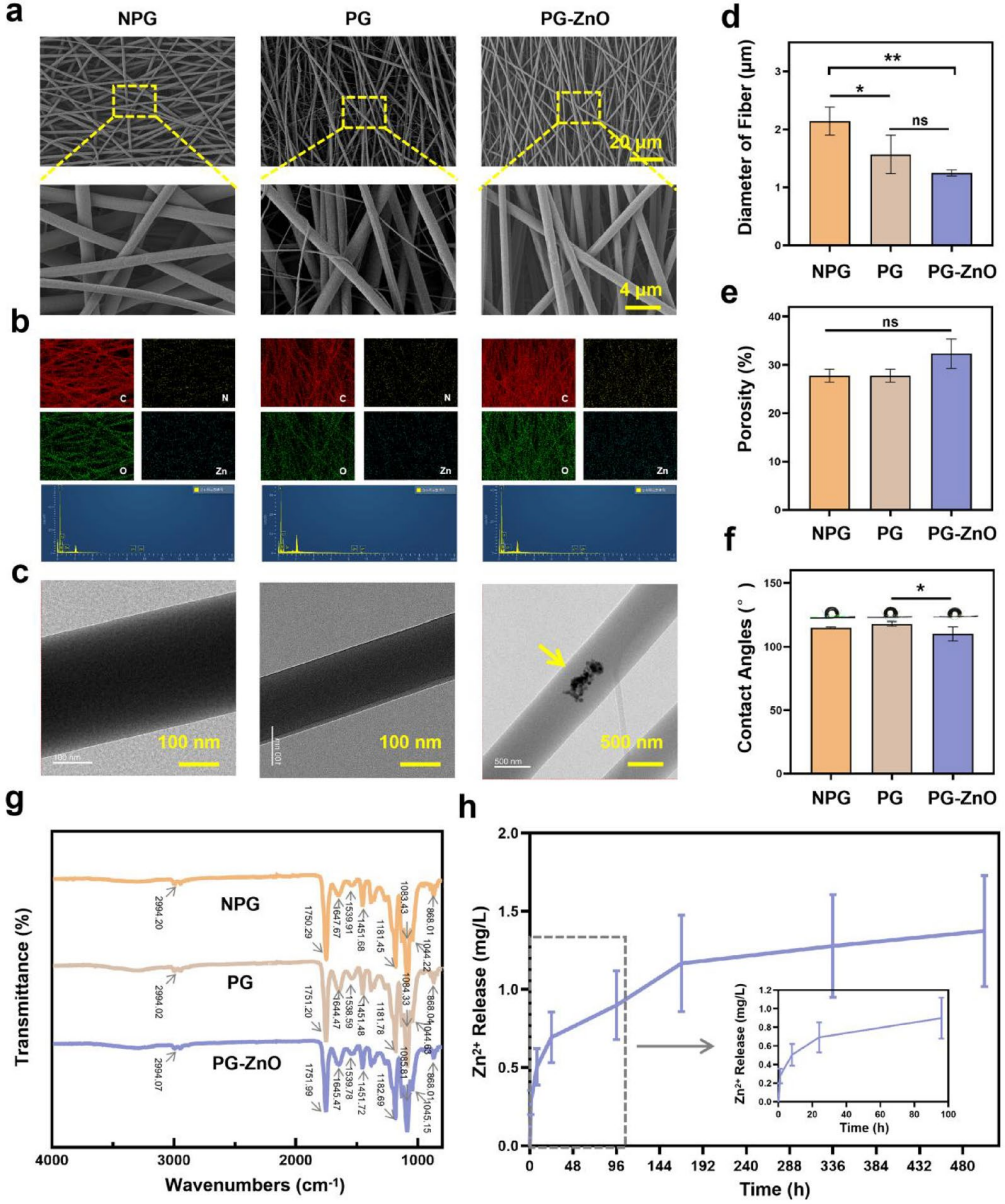
Characterization of membranes. **a**) SEM images and **b**) mapping of the membranes in each group. **c**) TEM images showing the fibre ultrastructure, with ZnO nanoparticles encapsulated within the fibres (yellow arrows). **d**) Fibre diameter and **e**) porosity analysis. **f**) Water contact angle. **g**) FTIR spectra of the membranes. **h**) Cumulative Zn²⁺ release curve (*p < 0.05, **p < 0.01)

Electrospun membranes effectively mimic the ECM and exhibit excellent biocompatibility and the ability to promote cell growth [13]. However, their mechanical strength is often insufficient to provide robust structural support for pelvic floor muscle repair, particularly under the sustained mechanical stress induced by abdominal pressure. Both PLA and PLLA possess favourable mechanical properties, making them suitable candidates for reinforcement [33, 34]. By blending these two polymers with Gel, which is a naturally derived biopolymer with high biocompatibility, NPG and PG membranes were fabricated by electrospinning. Additionally, based on the results of CCK-8 assay (using HUVECs), we chose to incorporate ZnO nanoparticles at a concentration of 5 mg/10 mL into the PG matrix to yield the PG-ZnO membrane (Fig. S1). SEM analysis (Fig. 1a) revealed that the three membranes exhibited continuous, bead-free, randomly oriented nanofibrous network structures with interconnected porous architectures. Notably, the substitution of PLA with PLLA did not affect the morphology or processability of the fibres. This ECM-mimetic network structure facilitates efficient cell adhesion, infiltration, and nutrient diffusion. Compared with those in the other two groups, the fibres in the PG-ZnO group had slightly rougher surfaces, indicating the incorporation of ZnO nanoparticles into the matrix. Elemental mapping (Fig. 1b) confirmed the uniform distribution of carbon (C), nitrogen (N), oxygen (O), and zinc (Zn) throughout the matrix, providing strong preliminary evidence that the ZnO nanoparticles were homogenously dispersed within the fibres. To further validate the spatial localization and physical state of ZnO, TEM was performed (Fig. 1c). TEM clearly revealed that the ZnO nanoparticles were embedded within the interior of the fibres rather than merely being adsorbed to the surface. This core-encapsulation structure is critical for enabling sustained and controlled release of Zn²⁺, thereby supporting prolonged anti-inflammatory activity.

Comparative analysis of the fibre diameter and porosity across the three groups of membranes revealed that the average fibre diameters of the PG membrane and PG-ZnO membrane were significantly smaller than that of the NPG membrane (Fig. 1d). This difference may stem from the greater crystallinity and piezoelectric responsiveness of the PLLA phase than the PLA phase. Consequently, under identical electric fields, the PLLA spinning solution undergoes greater tensile stress during jet spinning, resulting in finer fibres [35, 36]. Despite these differences in fibre diameter, no significant differences in porosity were observed among the groups (Fig. 1e). This consistency in pore structure may stem from a compensatory effect, wherein reduced fibre diameter is offset by denser fibre packing, thereby maintaining membrane porosity. Water contact angle measurements demonstrated that the contact angle of the PG-ZnO membrane (110.08 ± 5.40)° was slightly smaller than that of the PG membrane (Fig. 1f). This slight decrease can be attributed to the inherent hydrophilicity of the ZnO nanoparticles, which not only increases the surface roughness but also introduces additional hydrophilic sites, thereby promoting the spread and penetration of water [37]. Increased hydrophilicity is typically associated with favourable cell adhesion behaviour, providing a more suitable microenvironment for subsequent cellular activities [38].

The swelling characteristics of the membranes play a crucial role in the diffusion of nutrients and cells. Fig. S2a shows the swelling rate of the electrospun membranes in PBS for 72 h. Observations revealed that the PG-ZnO membrane exhibited obvious swelling behavior at 0.5 h, which was significantly higher than the other two groups. This was related to the slightly higher hydrophilicity of the PG-ZnO membrane compared to the other two groups. From 1 h onwards, the swelling rates of the PG group and the PG-ZnO group were significantly higher than that of the NPG group. After 24 h, the swelling rates of membranes gradually decreased, which might be related to the beginning of polymer degradation. The rapid initial swelling is conducive to the close adhesion of the membrane to the tissue and the diffusion of nutrients, while the subsequent decrease in swelling indicates that the material begins to undergo early remodeling. The degradation experiment results showed that the degradation rates of the PG membrane and the PG-ZnO membrane at all time points were significantly higher than those of the NPG membrane, but there was no significant difference between the two groups (Fig. S2b). This indicates that the PLLA itself is the main factor driving the degradation process, and the incorporation of ZnO nanoparticles did not further accelerate the degradation process. The NPG membrane had the slowest degradation rate because the PLA in this group had a lower crystallinity and lacked the sensitive hydrolysis characteristics of PLLA. This degradation characteristic enables the PG membrane and PG-ZnO membrane to gradually degrade after providing early mechanical support, providing space for the remodeling of new tissues.

FTIR analysis revealed characteristic absorption peaks in all three groups (Fig. 1g), including C = O stretching vibrations at approximately 1700–1800 cm⁻¹ and C-O-C stretching vibrations at approximately 1000–1300 cm⁻¹. The FTIR spectra of these two PLLA-based membranes (PG membrane and PG-ZnO membrane) were nearly superimposable, indicating that the incorporation of ZnO nanoparticles did not alter the fundamental chemical structure of the polymer matrix. Notably, no new vibrational bands attributable to covalent bonding were detected, suggesting that the interaction between ZnO and the polymer components is governed primarily by physical encapsulation or weak electrostatic forces rather than the formation of strong chemical bonds. As shown in Fig. 1h, the Zn²⁺ release curve exhibited a moderate burst release in the initial phase, followed by a sustained and progressive long-term release phase. It is noteworthy that within 3 weeks, the cumulative release concentration of Zn^2+^ was approximately 0.82–0.84 µg/mL, which was much lower than the toxicity threshold (> 2 µg/mL), and was at the same order of magnitude as the physiological plasma zinc concentration (0.7–1.2 µg/mL). The initial burst release likely originates from loosely bound or surface-exposed ZnO nanoparticles, whereas the subsequent sustained release stems from the gradual degradation of ZnO embedded within the fibres and the diffusion of Zn²⁺. This controlled and prolonged Zn²⁺ release behaviour is essential for modulating the local immune microenvironment at the implantation site, enabling long-lasting anti-inflammatory effects while minimizing the risk of cytotoxicity associated with rapid ion release.

### Piezoelectric and mechanical properties of membranes

**Fig. 2:**
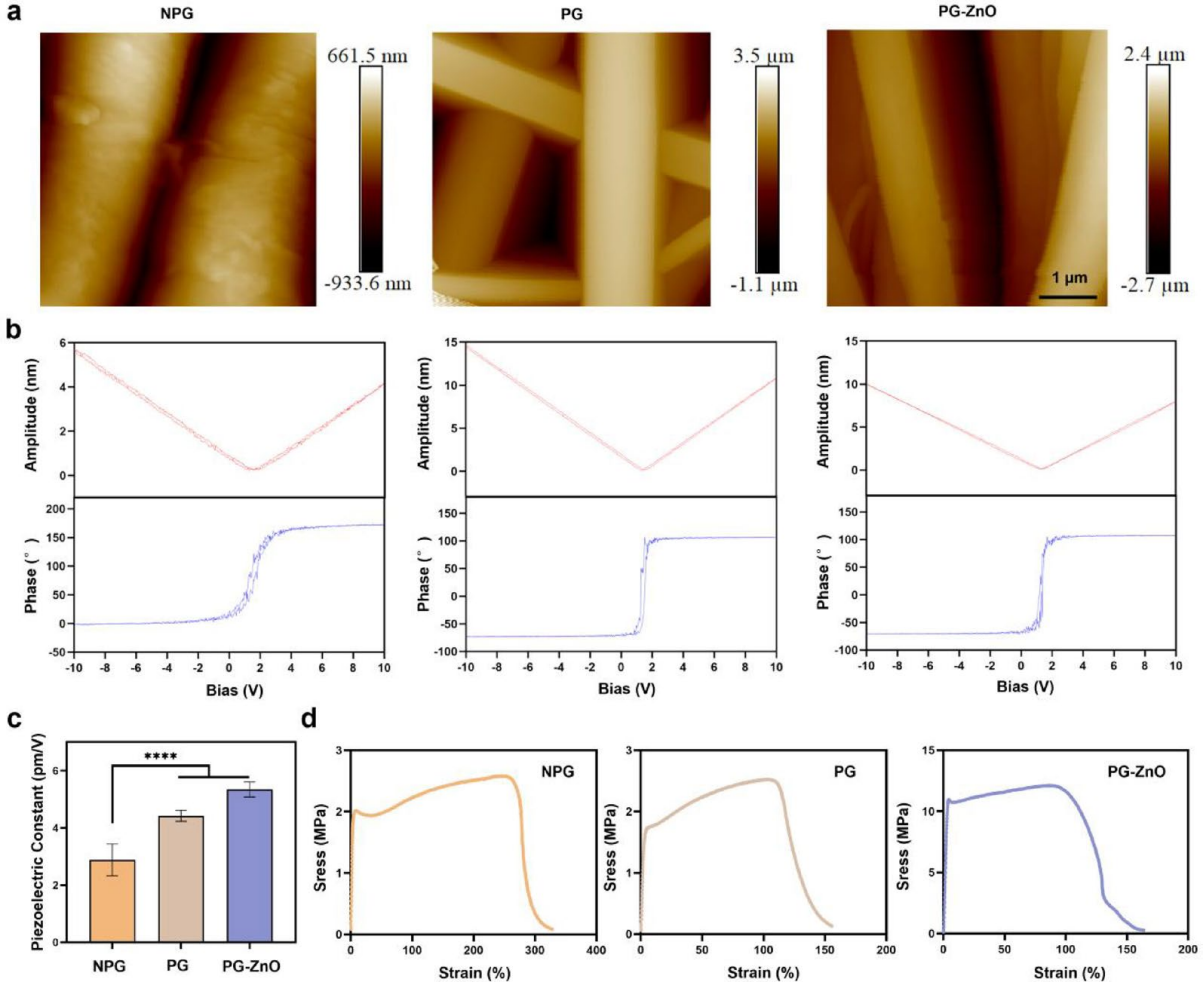
Piezoelectric and mechanical properties of the membranes. **a**) AFM images of the fibres. **b**) Local piezoelectric response measured by PFM. **c**) Quantitative comparison of the *d₃₃*. **d**) Tensile stress–strain curves and mechanical parameters of the three membrane groups (*p < 0.05, **p < 0.01)

The surface microstructures of the membranes were characterized by AFM, as shown in Fig. 2a. Significant differences in surface morphology were observed among the three groups of membranes. The fibres of the NPG membrane exhibited relatively smooth surfaces with an average roughness of 207 nm but poor flatness. In contrast, the surface roughness values of the fibres of the PG membrane and PG-ZnO membrane increased to 606 nm and 620 nm, respectively. This increase was primarily attributed to more pronounced surface topographical features. The incorporation of ZnO nanoparticles further contributed to surface changes resulting from the higher crystallinity of PLLA. The incorporation of ZnO nanoparticles further exacerbated surface irregularities. However, the increase in the surface roughness of the fibres was only marginal compared with that of the PG membrane, indicating that the ZnO nanoparticles were primarily embedded within the fibre structure rather than accumulated on the surface. This substantial increase in surface roughness not only reflects compositional changes but also provides favourable physical signals for cell adhesion, thereby promoting tissue repair [39, 40]. Local piezoelectric deformation was assessed via PFM (Fig. 2b), and the corresponding piezoelectric coefficient *d₃₃* values were extracted from the scanned regions. The static *d₃₃* values of the PG membrane and PG-ZnO membrane were 4.42 ± 0.19 pm/V and 5.34 ± 0.26 pm/V, respectively, which were significantly greater than that of the NPG membrane (2.88 ± 0.55 pm/V) (Fig. 2c). As shown in Fig. S3, the PG membrane and the PG-ZnO membrane, especially the PG-ZnO membrane, exhibited excellent electromechanical conversion performance. With the increase of the applied pressure, the peak output voltage significantly increased. Moreover, after a continuous 10-second mechanical stimulation cycle, the membrane still maintained a relatively stable voltage output, further verifying its outstanding piezoelectric performance. These results indicate that the enhanced piezoelectric response primarily stems from the high crystallinity of PLLA, whereas the addition of ZnO nanoparticles has a minimal effect on the piezoelectric properties of the membrane. This significant increase in piezoelectric performance enables both the PG membranes and the PG-ZnO membranes to convert physiological mechanical stimuli from pelvic floor muscle tissues into endogenous electrical signals, providing sustained bioelectrical stimulation crucial for tissue regeneration [16, 41].

Assessment of the mechanical properties revealed significant differences in tensile behaviour among the groups (Fig. 2d). Stress–strain curves revealed that the ductility of the NPG membrane was greatest among all the materials, with elongation at break reaching 273.33%, but its ultimate tensile strength was relatively low (2.58 MPa). Compared with the NPG membrane, the PG membrane did not significantly differ in terms of ultimate tensile strength but did show reduced ductility. In contrast, the PG-ZnO membrane demonstrated superior comprehensive mechanical properties: the ultimate tensile strength significantly increased to 12.11 MPa while sufficient ductility was maintained. Furthermore, through conducting the tensile cycle test, it can be observed that the tensile strength of the PG-ZnO membrane is between that of the NPG membrane and the PG membrane. After 5 cycles, it still maintains stable stress (Fig. S4). Studies have shown that the elastic modulus of vaginal tissues in pre-menopausal normal women is 6.65 ± 1.48 MPa, while that of patients with mild POP is 9.45 ± 0.70 MPa [42]. The ultimate tensile strength of the PG-ZnO membrane (12.11 MPa) is within the high value range of the above physiological range and can better simulate the mechanical properties of the muscle and fascia system in the body, providing stable support for the tissues. This improvement is primarily attributed to interfacial interactions between the zinc oxide nanoparticles and the polymer chains, which increase the stiffness and strength [43, 44].

The excellent mechanical properties and water absorption, significant piezoelectric response, and increased surface roughness make the PG-ZnO membrane an ideal material for pelvic floor repair. They provide robust mechanical support under physiological loads while stimulating cellular activity through in situ generation of bioelectric signals. Increased surface roughness facilitates cell adhesion and tissue integration. These properties synergistically promote the repair of pelvic floor muscles or other soft tissues.

### In vitro cell adhesion and tube formation

**Fig. 3:**
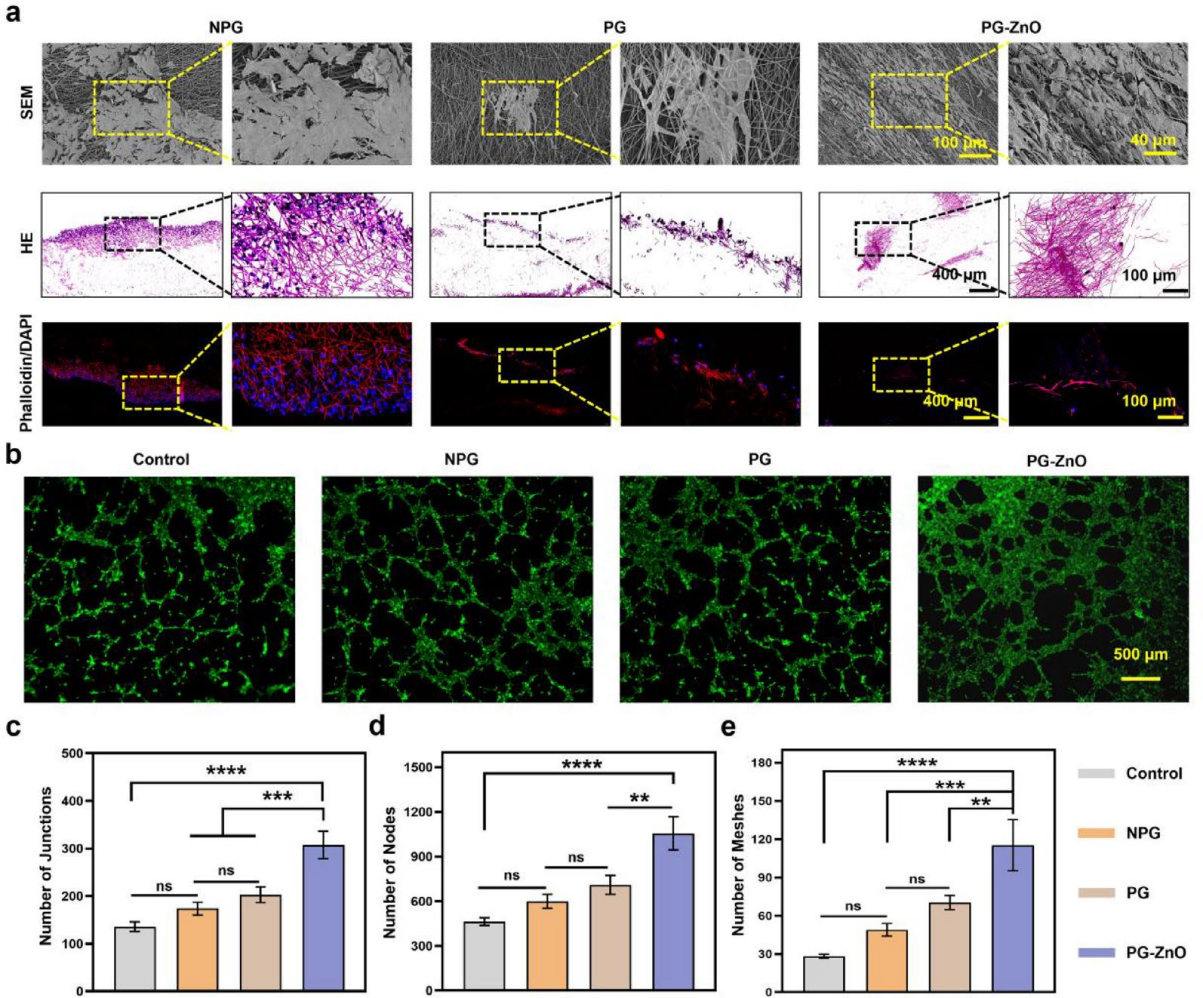
Cell adhesion and tube formation ability of the membranes. **a**) H&E staining, immunofluorescence staining, and SEM images. **b**) Tube formation assay. **c**) Number of junctions. **d**) Number of meshes. **e**) Number of nodes (*p < 0.05, **p < 0.01)

To evaluate the cell adhesion properties of the electrospun membranes, HUVECs were cultured on the membranes for one week. H&E staining, immunofluorescence staining, and SEM revealed that the cells grew smoothly along the fibre structures and maintained good cellular viability across all three membranes. These results indicate that the incorporation of ZnO does not compromise the inherent cell adhesion properties of the membranes and that all the membranes provide a favourable microenvironment for cell adhesion and growth (Fig. 3a).

Angiogenesis plays a critical role in tissue repair by providing more efficient nutrient delivery pathways to damaged areas and effectively transporting prorepair factors to accelerate tissue repair [45, 46]. Tube formation assays revealed that HUVECs cultured with the extract liquid of the PG-ZnO membrane formed the most complete tubular network within 4 h (Fig. 3b). The number of junctions reached 307.33 ± 28.99 per field of view, which was significantly greater than the numbers in the PG group (203.00 ± 16.52) and NPG group (173.67 ± 13.50). Node analysis revealed that the number of nodes (1056.00 ± 110.64 per field of view) and meshes (115.33 ± 20.00 per field of view) was markedly greater in the PG-ZnO group than in the other two groups (Fig. 3c–e). These results indicate that Zn^2+^ release effectively enhances angiogenesis, potentially through the upregulation of angiogenesis-related factors.

### Biocompatibility and anti-inflammatory

**Fig. 4:**
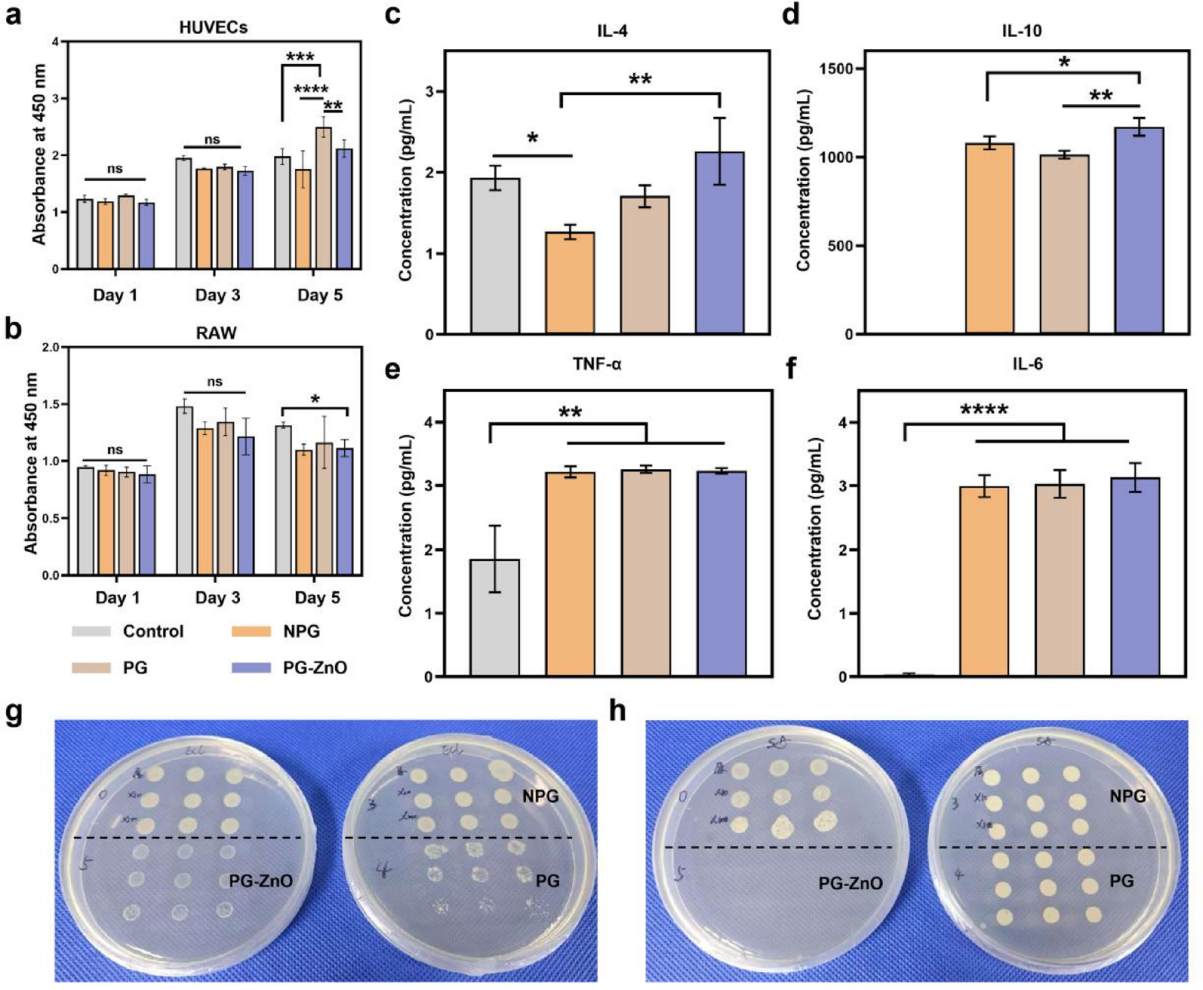
Biocompatibility, anti-inflammatory and antibacterial properties. **a**) CCK-8 assay of HUVECs. **b**) CCK-8 assay of RAW 264.7 cells. **c**) IL-4 and **d)** IL-10 concentrations determined by ELISA. **e**) TNF-α and **f**) IL-6 concentrations determined by ELISA. **g**) Antibacterial effects against *Escherichia coli*. **h**) Antibacterial effects against *Staphylococcus aureus* (*p < 0.05, **p < 0.01)

Biocompatibility was evaluated using HUVECs and RAW 264.7 cells. None of the membranes exhibited cytotoxicity towards either cell type, confirming that these membranes are biocompatible (Fig. 4a–b). With respect to RAW 264.7 cells, the PG-ZnO group showed mild inhibition of cell proliferation, yet the cellular activity remained normal on Day 5 (Fig. 4b). This specific phenomenon may correspond to its anti-inflammatory effect, as moderate suppression of excessive immune cell activation is often associated with anti-inflammatory responses.

The anti-inflammatory properties represent the immunomodulatory characteristics of the membranes. ELISA results (Fig. 4c–f) showed that while the expression of the proinflammatory factors TNF-α and IL-6 was comparable across all the groups, the expression of the two anti-inflammatory factors, IL-4 and IL-10, was significantly greater in the PG-ZnO group than in the other groups. Thus, The PG-ZnO group markedly outperformed the other two groups. Because the PG-ZnO membrane preferentially upregulates the expression of anti-inflammatory cytokines (IL-4 and IL-10), while under the same conditions, the inhibition of pro-inflammatory cytokines TNF-α and IL-6 is not significant. Therefore, ZnO nanoparticles may exert their effects by promoting the secretion of anti-inflammatory factors. Previous studies have shown that Zn^2+^ can inhibit the activation of the NF-κB signaling pathway, induce macrophages to polarize towards the M2 phenotype, and thereby exert anti-inflammatory effects [47, 48]. Notably, this specific immunomodulatory approach in the PG-ZnO group enhanced tissue repair capacity while preserving fundamental inflammatory defence functions, thereby creating an ideal immune microenvironment for pelvic floor repair. These findings also offer new insights for the design of immunomodulatory strategies in tissue engineering materials.

### Antibacterial properties

The qualitative antibacterial experiments revealed that the PG-ZnO membrane exhibited significant antibacterial activity against both *Escherichia coli* and *Staphylococcus aureus* (Fig. 4g–h). The colony counts also confirmed the antibacterial effect of the PG-ZnO membrane (Fig. S5). This powerful antibacterial activity is primarily attributed to the sustained release of Zn^2+^, which exerts antibacterial effects through multiple mechanisms, including the disruption of bacterial membrane integrity and induction of oxidative stress [49]. The PG-ZnO membrane maintains its biocompatibility while exerting antibacterial effects, a property that provides effective protection against postimplantation infections during pelvic floor repair procedures.

### In vivo experiments

The pathogenesis of POP involves a complex interaction of multiple factors, including pregnancy, ageing, and mechanical injury. Currently, mature animal models that can accurately simulate the pathophysiological processes of POP are lacking, which significantly limits the development of its repair materials and mechanistic research [32, 50]. To simulate the repair processes of POP, this study established a full-thickness abdominal wall defect model in female SD rats. This model is one of the commonly used and frequently selected models in current pelvic floor repair research [32, 51, 52]. As shown in Fig. S6, a circular 1 cm diameter abdominal wall muscle defect was created, and electrospun membranes were implanted into this defect (Day 0). Since it can adhere well to the damaged tissue area, no additional fixation measures are required. Tissue samples were collected at Weeks 2 and 4. Local inflammatory responses were assessed using HE staining, while collagen deposition areas were quantified via Masson staining to evaluate the repair efficacy of the PG-ZnO membrane.

On Week 2, the control group exhibited a distinct purulent mass at the surgical site, which felt firm to the touch and emitted a foul odour. In contrast, the surgical sites of the other three groups showed no significant purulent discharge or odour, with only mild local redness and swelling present (Fig. S7). This finding indicates that in the absence of electrospun membrane implantation, the defect areas, which lack tissue barrier protection, are susceptible to bacterial colonization and subsequent suppurative infection. The physical barrier function of the electrospun membranes can preliminarily block external pathogen invasion, providing fundamental protection for wound healing.

**Fig. 5:**
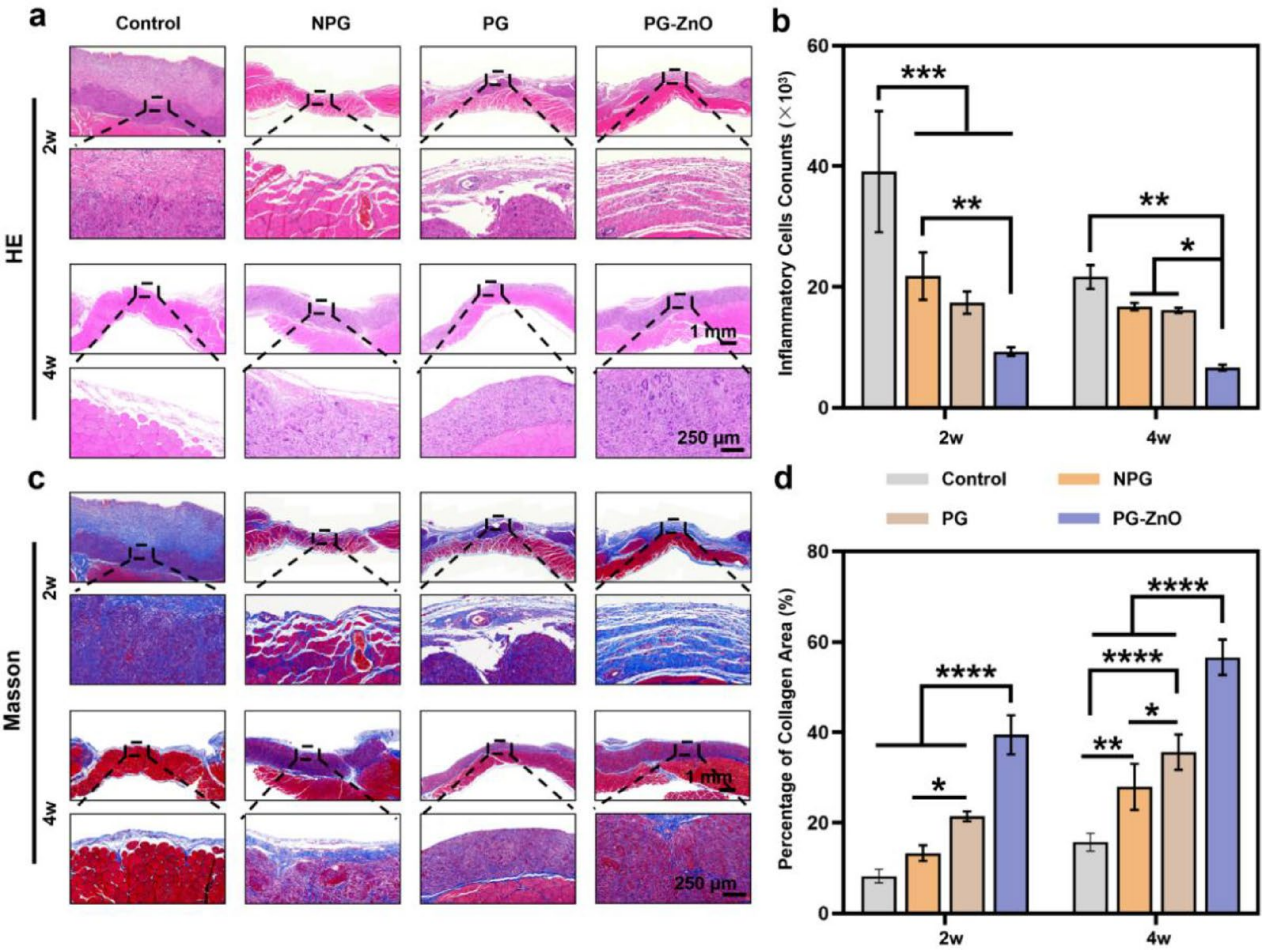
In vivo histological analysis of tissue regeneration. **a**) H&E staining images. **b**) Quantitative analysis of inflammatory cell infiltration. **c**) Masson’s trichrome staining images. **d**) Quantitative analysis of the collagen-positive area (*p < 0.05, **p < 0.01)

H&E staining revealed extensive inflammatory cell infiltration in the control group at Week 2, characterized by a disorganized cell arrangement and localized tissue necrosis, with slow tissue repair progression. Compared with the control group, the NPG and PG groups had slightly lower inflammatory cell counts but maintained relatively high infiltration levels. In contrast, the inflammatory cell density significantly decreased in the PG-ZnO group, with only a small number of scattered inflammatory cells visible at the tissue margins. At Week 4, although inflammatory responses diminished across all groups, but the PG-ZnO group exhibited markedly lower inflammatory cell infiltration than the other groups (Fig. 5a–b). These results suggest that the PG-ZnO membrane has a sustained anti-inflammatory effect, which is attributed to the slow release of Zn²⁺. In contrast, the NPG membrane demonstrated limited anti-inflammatory efficacy because of the absence of Zn^2+^ regulation, further confirming the pivotal role of Zn^2+^ in the anti-inflammatory function of the PG-ZnO membrane.

Collagen deposition is a critical step in tissue repair [53]. Masson’s trichrome staining (Fig. 5c–d) revealed that compared with the other three groups, the PG-ZnO group exhibited a significantly greater proportion of collagen-positive areas at Week 2. Compared with the NPG group, the PG group, benefiting from the piezoelectric effect of the membrane that converts mechanical stimuli into electrical signals, stimulated fibroblast proliferation and collagen synthesis, resulting in greater collagen deposition. The control and NPG groups showed only sparse, scattered collagen fibres at the wound margins, which were arranged in a disordered pattern. At Week 4, the collagen-positive area was further increased in the PG-ZnO group, with collagen fibres arranged in bundles and largely integrated with the surrounding normal muscle tissue. Compared with the PG-ZnO group, the PG group had a significantly smaller collagen-positive area, while the control and NPG groups maintained low collagen deposition levels.

**Fig. 6:**
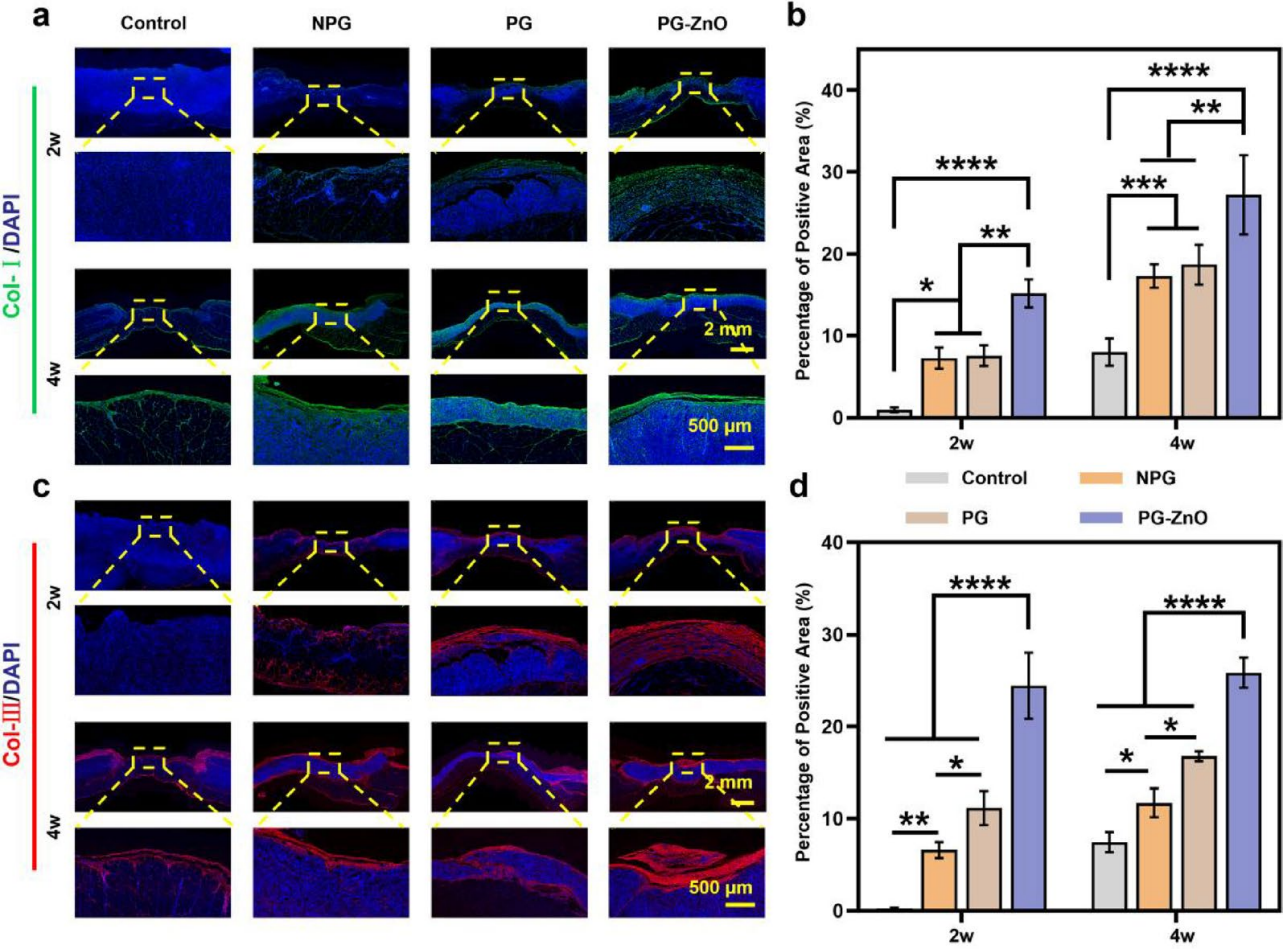
Immunofluorescence staining of collagen deposition. **a**) Immunofluorescence images of Col-I; **b**) Quantification of the relative Col-I-positive areas. **c**) Immunofluorescence images of Col-III; **d**) Quantitative analysis of the relative Col-III-positive areas (*p < 0.05, **p < 0.01)

Collagen synthesis was analysed using immunofluorescence quantification of type I collagen (Col-I) and type III collagen (Col-III). As shown in Fig. 6a–b, during Week 2, compared with the other three groups, the PG-ZnO group had a significantly greater percentage of Col-I-positive area (15.17%±1.70%). The proportions in the NPG and PG groups were not significantly different but were greater than those in the control group. This trend persisted at 4 weeks post-implantation, with the Col-I positive area in the PG-ZnO group reaching 27.22%±4.84%, which was the greatest among all the groups. For Col-III (Fig. 6c–d), the PG-ZnO group also had the greatest positive area percentage (24.43%±3.59%) at Week 2, significantly exceeding that of the other groups. The PG group (11.15%±1.85%) outperformed the NPG group (6.59%±0.86%), and the areas in both groups were markedly greater than that in the control group (0.25%±0.08%). This hierarchical pattern remained consistent at Week 4.

These results indicate that the deposition level of Col-III was significantly higher in the PG group compared to the NPG group. This finding suggests that the piezoelectric properties of PLLA may promote the synthesis of Col-III. In contrast, the synergistic “piezoelectric+anti-inflammatory” effect in the PG-ZnO group simultaneously enhanced the deposition efficiency of both Col-I and Col-III. The anti-inflammatory properties of the material create a stable microenvironment for sustained Col-I synthesis by reducing inflammation-mediated collagen degradation [54, 55]. As it is the primary component of mature collagen, high Col-I expression further ensures structural integrity during pelvic floor tissue repair.

**Fig. 7:**
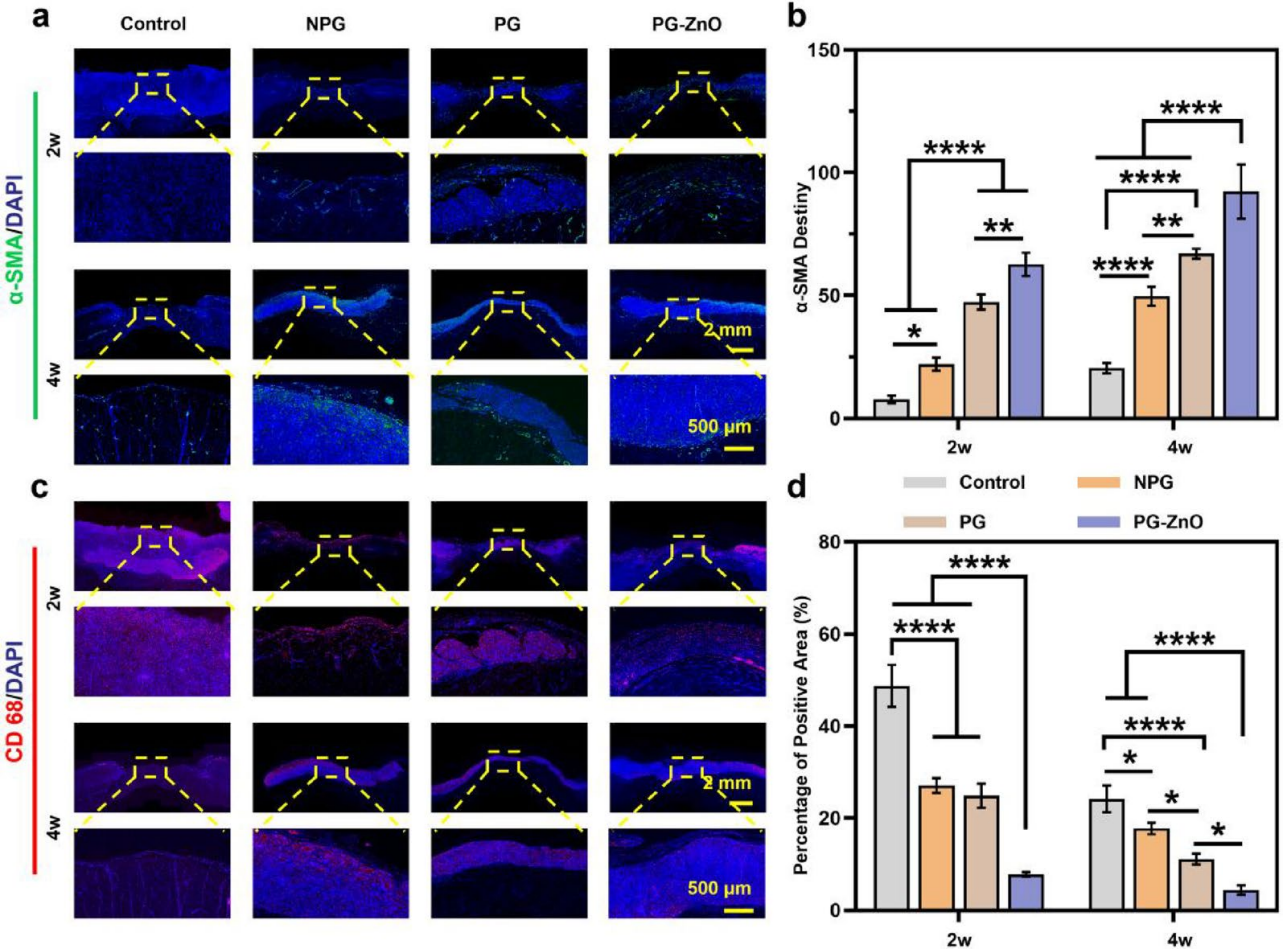
Immunofluorescence staining of α-SMA and CD68. **a**) Immunofluorescence images of α-SMA; **b**) Quantification of α-SMA density. **c**) Immunofluorescence images of CD68; **d**) Quantitative analysis of the relative CD68-positive areas (*p < 0.05, **p < 0.01)

The α-SMA marker was selected to evaluate angiogenesis. At Week 2, compared with the other three groups, the PG-ZnO group exhibited significantly greater quantitative positive vascular density. The NPG and PG groups were not significantly different, but both were superior to the control group. At Week 4, the vascular density of the PG-ZnO group was the greatest, while that of the PG group significantly exceeded that of the NPG group (Fig. 7a–b). The CD31 immunofluorescence staining at Week 4 confirmed that the positive area in the PG-ZnO group was significantly higher than that in the NPG group and the PG group (Fig. S8). These findings suggest that early angiogenesis primarily relies on the physical barrier function of the membranes, which protects vascular endothelial cells from infection-induced damage. Later, the piezoelectric effect regulates endothelial cell proliferation and migration through electrical signalling [56]. The PG-ZnO membrane may reduce tissue oxidative stress because of the anti-inflammatory properties of Zn^2+^, thereby promoting macrophage polarization and enhancing endothelial cell migration and vascular formation [57].

**Fig. 8:**
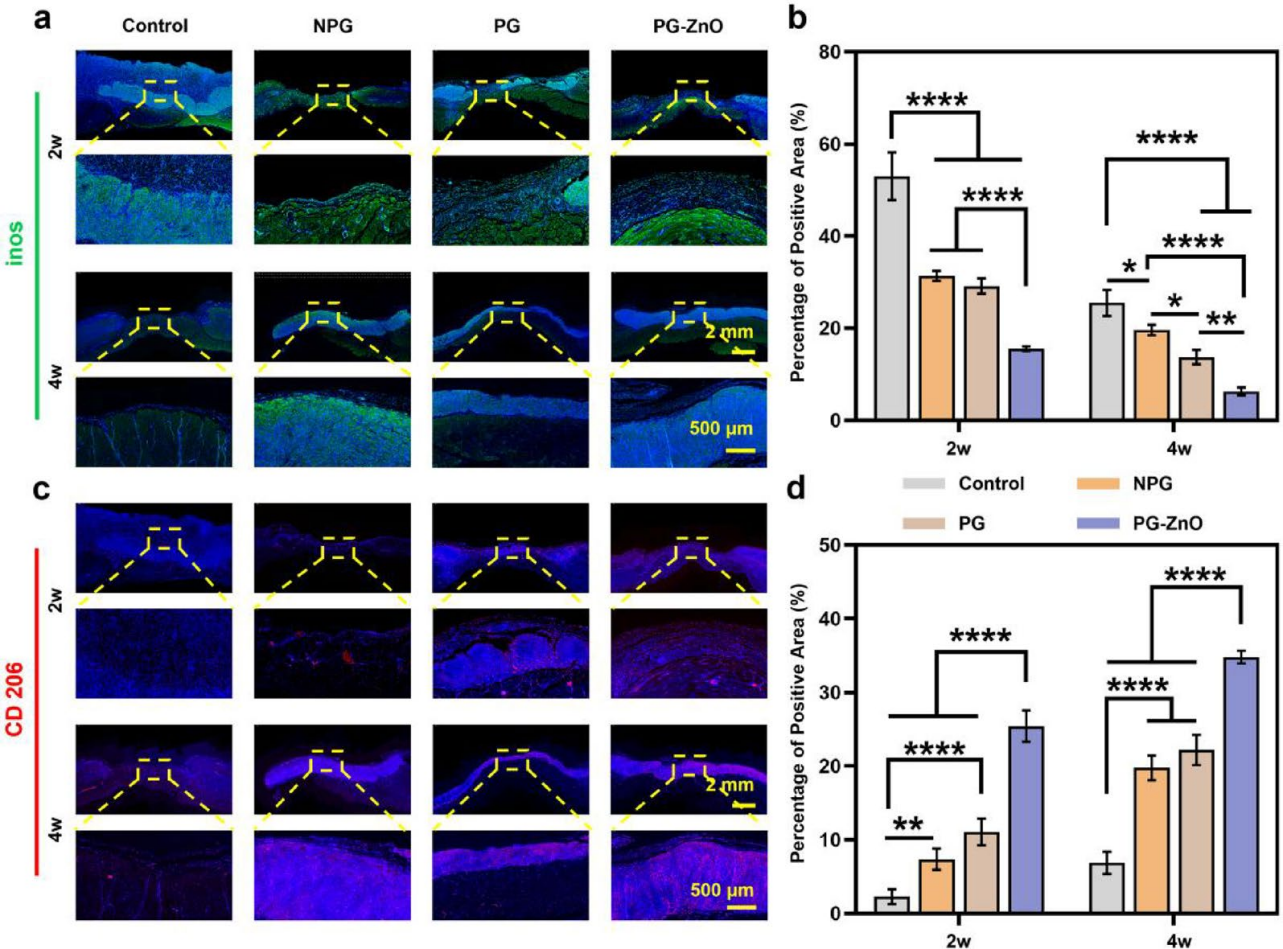
Immunofluorescence staining of macrophage polarization markers. **a**) Immunofluorescence images of iNOS; **b**) Quantitative analysis of the relative iNOS-positive areas. **c**) Immunofluorescence images of CD206; **d**) Quantification of the relative CD206-positive areas (*p < 0.05, **p < 0.01)

The in vivo immunomodulatory capacity of the electrospun membranes was analysed using CD68, iNOS, and CD206. The expression of CD68, a marker of total macrophages, was significantly lower in the PG-ZnO group than in the other groups at Week 2. By Week 4, compared with the NPG group, the PG-ZnO group maintained the smallest positive area, and the PG group had a smaller positive area than the NPG group (Fig. 7c–d), indicating that the piezoelectric properties of the PLLA may also possess immunomodulatory capabilities during the late stage of tissue repair. M1 and M2 macrophages were subsequently labelled separately. iNOS serves as a marker of M1 macrophages, reflecting proinflammatory capacity, while CD206 is a marker of M2 macrophages, indicating anti-inflammatory effects. At Week 2, the control group had the greatest iNOS-positive area, while the PG-ZnO group had the smallest area. This trend persisted at Week 4, but the positive staining area of the PG group began to decrease and was smaller than that of the NPG group (Fig. 8a–b). These results suggest that the piezoelectric effect may suppress the proinflammatory capacity of macrophages during the late phase of tissue repair. At all time points, the PG-ZnO group exhibited the largest CD206-positive area. Compared with the control group, both the NPG and PG groups presented greater positive areas, although no significant difference was observed between these two groups (Fig. 8c–d). These findings suggest that piezoelectric properties may not play a key role in the in vivo anti-inflammatory effects, with anti-inflammatory activity likely mediated solely through zinc ion release. The results above indicate that the electrospun membranes can reduce macrophage infiltration through a physical barrier, whereas Zn²⁺ in the PG-ZnO group further significantly inhibited M1 phenotype differentiation and promoted M2 phenotype differentiation by regulating macrophage polarization signalling pathways. Additionally, during the late stages of in vivo pelvic floor repair, the piezoelectric effect of the PG-ZnO membrane may help alleviate macrophage infiltration and establish a favourable anti-inflammatory microenvironment for pelvic floor repair.

## Conclusion

This study innovatively designed a immunomodulatory piezoelectric master electrospun membrane. Uniformly incorporating ZnO nanoparticles into fibres with piezoelectric properties increases the hydrophilicity of the electrospun membrane to provide a more favourable adhesion interface while offering reliable mechanical support. This electrospun membrane also enables sustained and controlled release of Zn²⁺. In vitro experiments demonstrated that this electrospun membrane significantly enhances the tube-forming capacity of HUVECs while exhibiting anti-inflammatory and powerful antibacterial properties. When this membrane was applied to rat abdominal wall muscle defects, its piezoelectric and anti-inflammatory properties synergistically regulated vascular endothelial cell proliferation and migration while promoting macrophage polarization and collagen synthesis. Consequently, this immunomodulatory piezoelectric master electrospun membrane has promising application potential and clinical translational value in pelvic floor repair.

## Electronic Supplementary Material

Below is the link to the electronic supplementary material.

Supplementary material 1.

## Data Availability

All data generated or analysed during this study are included in this article.
