# Supplementary material for: Immunomodulatory piezoelectric master electrospun membranes for pelvic floor repair
Source: J Nanobiotechnology. 2026 May 22;24:669. doi: 10.1186/s12951-026-04582-0 (PMC13377707; doi:10.1186/s12951-026-04582-0)
Supplement: Supplementary file 1 — Supplementary material 1. [file 12951_2026_4582_MOESM1_ESM.docx]

Supporting Information


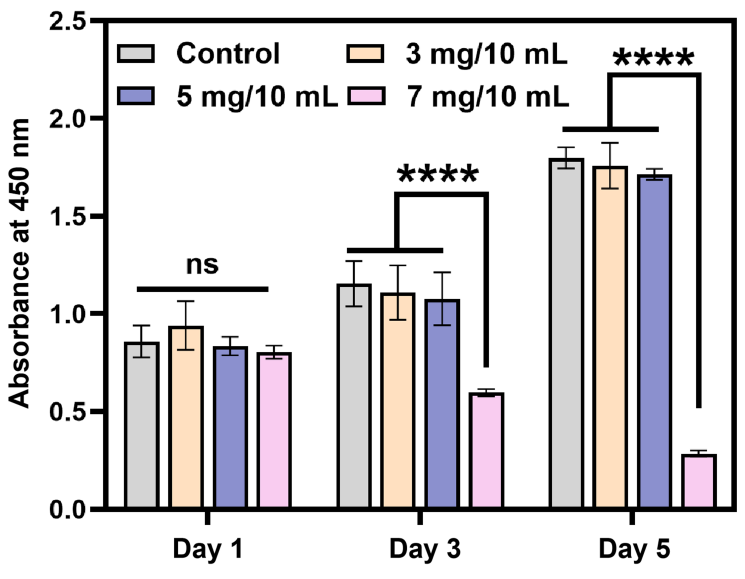


**Fig. S1** CCK-8 assay of the electrospun membranes with different ZnO contents.


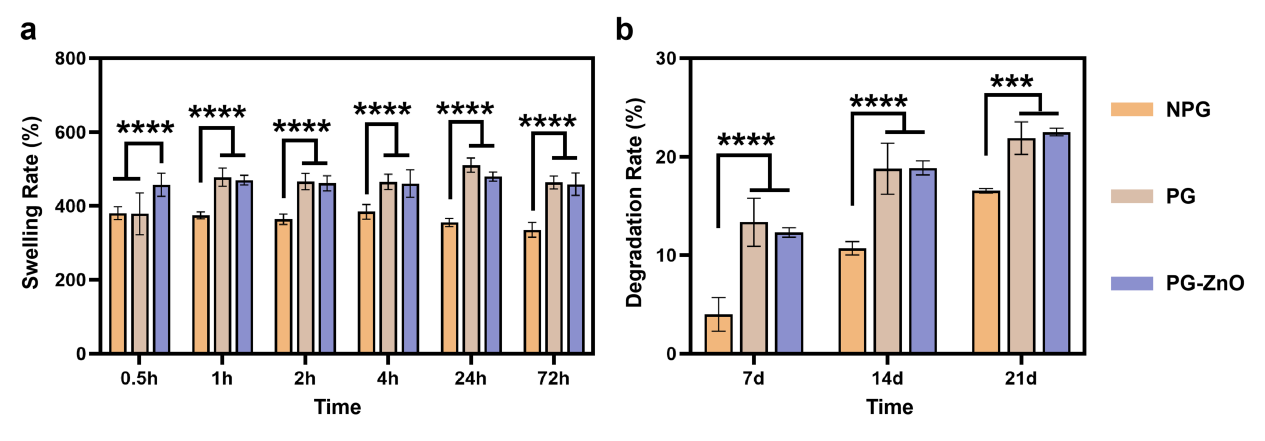


**Fig. S2** Swelling rate and degradation rate of the electrospun membranes. a) Swelling rate of the electrospun membranes; b) Degradation rate of the electrospun membranes (**p* < 0.05, ***p* < 0.01).


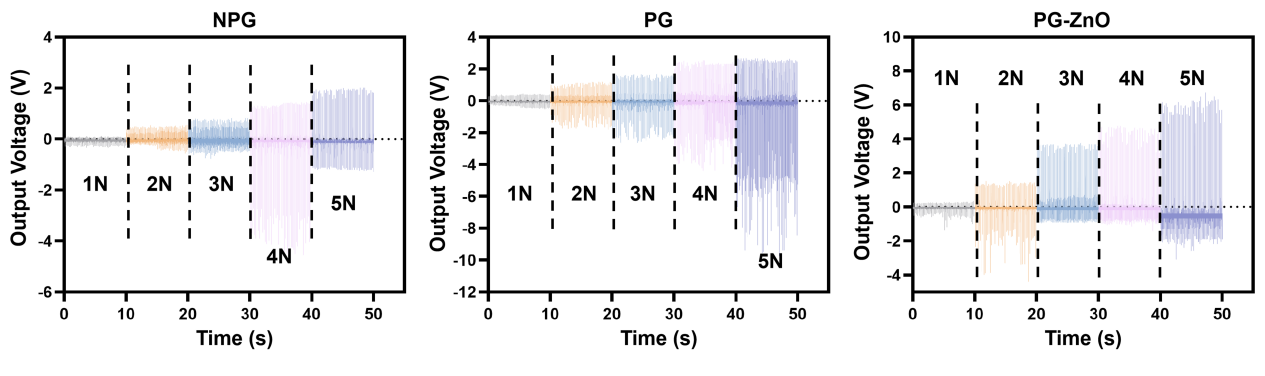


**Fig. S3** The output voltage of the electrospun membranes.


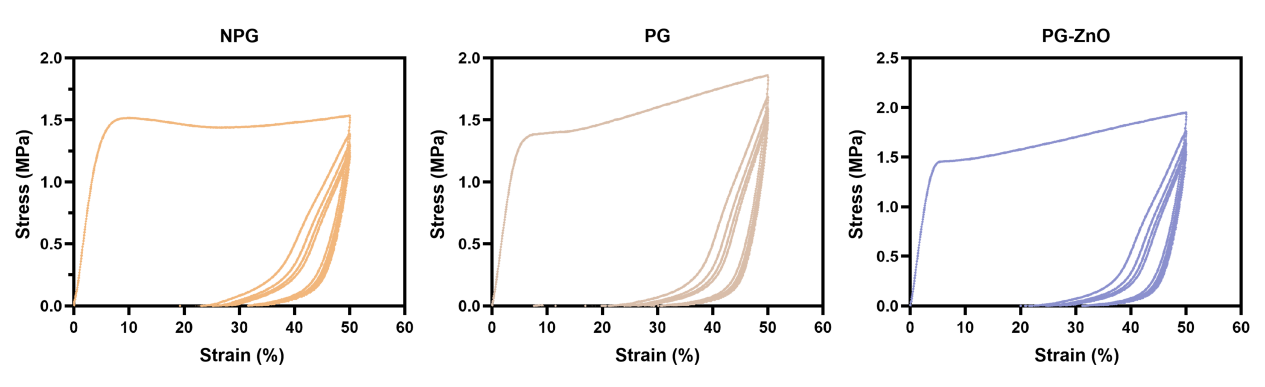


**Fig. S4** Tensile cycle tests of the electrospun membranes.


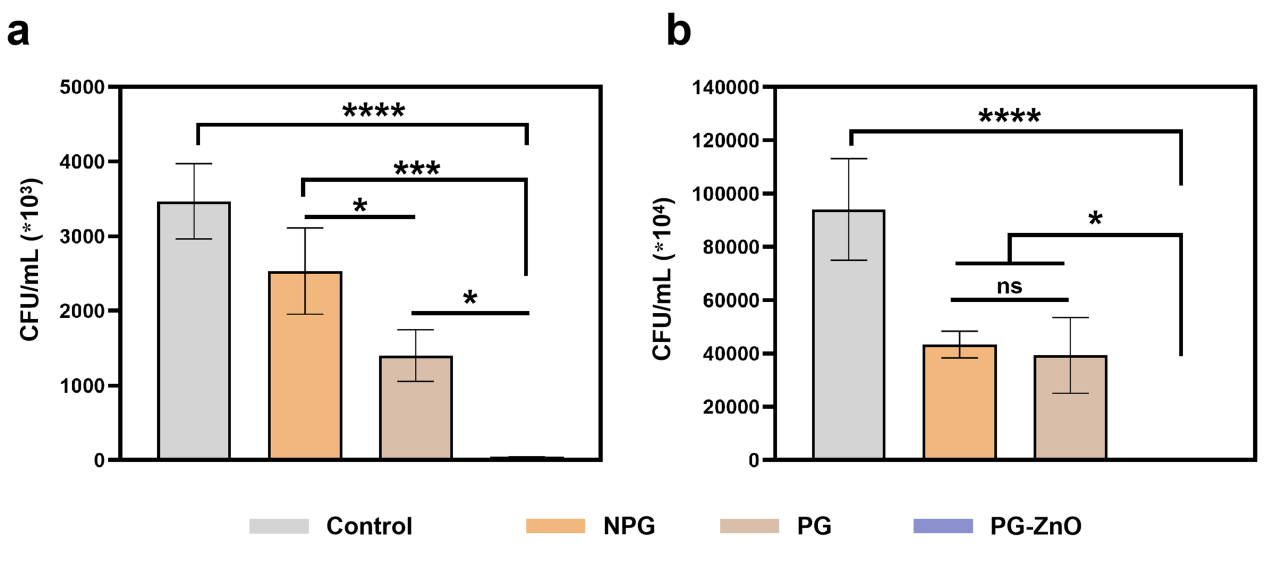


**Fig. S5** Antibacterial properties. a) The colony count of *Escherichia coli*; b) The colony count of *Staphylococcus aureus* (**p* < 0.05, ***p* < 0.01).


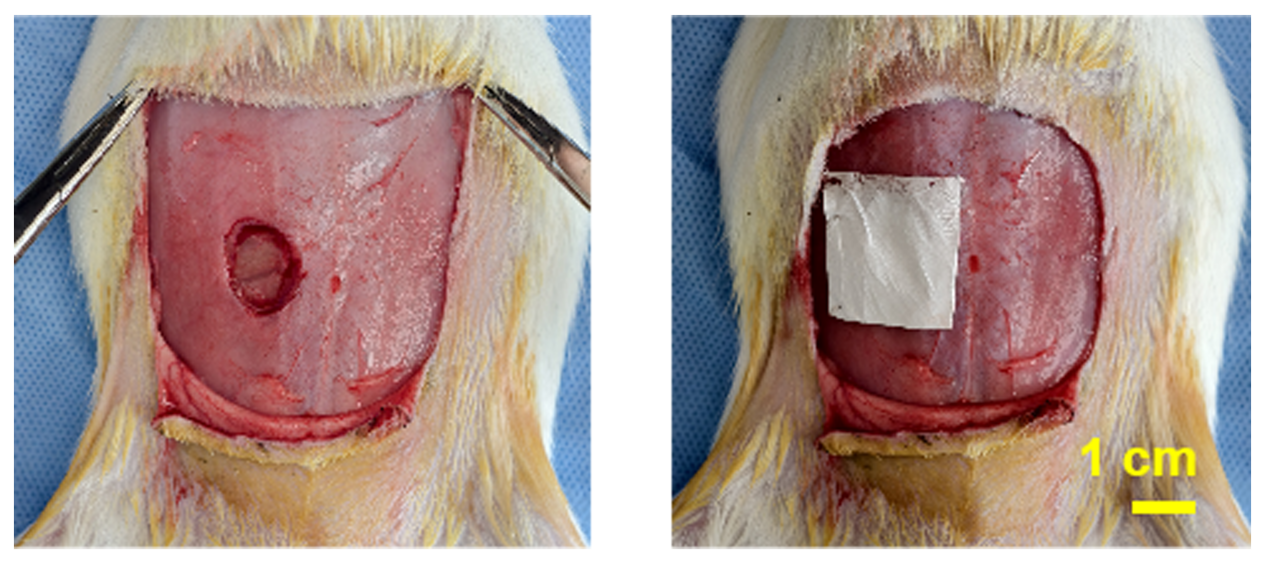


**Fig.S6** Before and after the placement of the electrospun membranes.


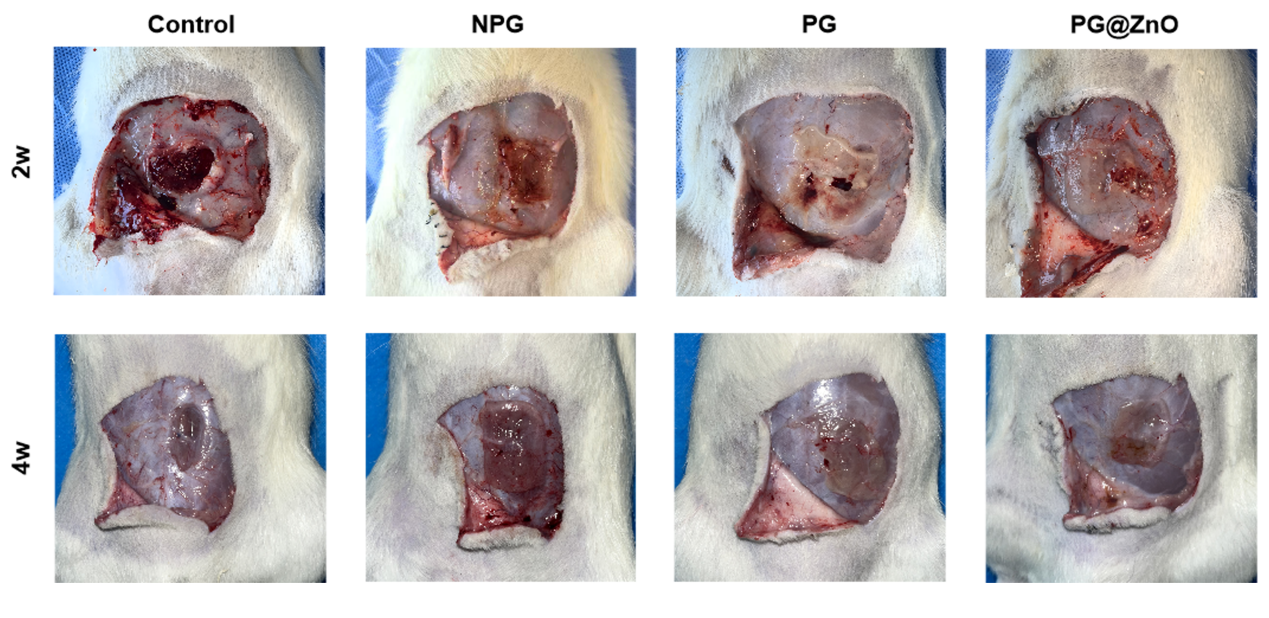


**Fig.S7** Healing outcomes in different treatment groups were assessed on Weeks 2 and 4.


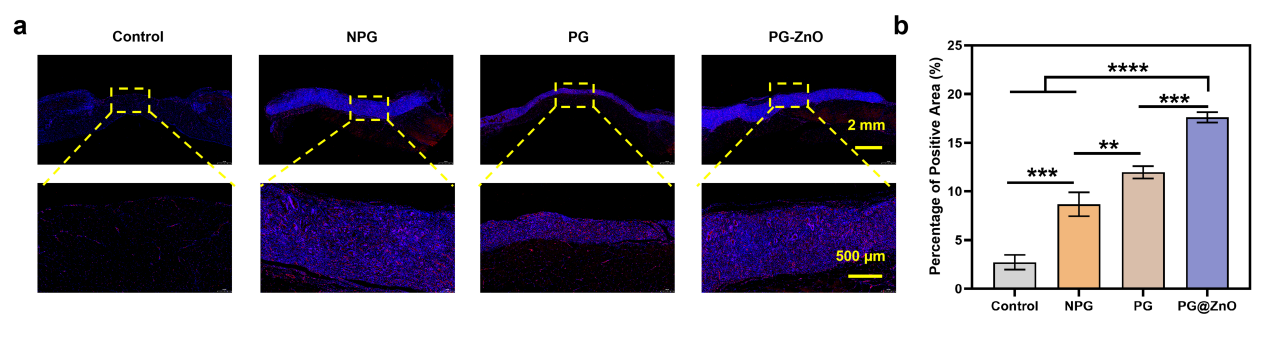


**Fig. S8** Immunofluorescence staining of CD31. a) Immunofluorescence images of CD31; b) Quantitative analysis of the relative CD31-positive areas (**p* < 0.05, ***p* < 0.01).
